# Ataxin-3 and Its E3 Partners: Implications for Machado–Joseph Disease

**DOI:** 10.3389/fneur.2013.00046

**Published:** 2013-05-06

**Authors:** Thomas M. Durcan, Edward A. Fon

**Affiliations:** ^1^Department of Neurology and Neurosurgery, Centre for Neuronal Survival and McGill Parkinson Program, Montreal Neurological Institute, McGill UniversityMontreal, QC, Canada

**Keywords:** parkin, ataxin-3, CHIP, Machado–Joseph disease, Parkinson’s disease, polyglutamine expansion

## Abstract

Machado–Joseph disease (MJD) is the most common dominant inherited ataxia worldwide, caused by an unstable CAG trinucleotide expansion mutation within the *SCA3* gene resulting in an expanded polyglutamine tract within the ataxin-3 protein. Ataxin-3 functions as a deubiquitinating enzyme (DUB), within the Ub system and whilst many DUBs are known to partner with and deubiquitinate specific E3-Ub ligases, ataxin-3 had no identified E3 partner until recent studies implicated parkin and CHIP, two neuroprotective E3 ligases. MJD often presents with symptoms of Parkinson disease (PD), which led to identification of parkin as a novel E3-Ub ligase whose activity was regulated by ataxin-3-mediated deubiquitination. Findings from these studies also revealed an unexpected convergence upon the E2-Ub-conjugating enzyme in the regulation of an E3/DUBenzyme pair. Moreover, mutant but not wild-type ataxin-3 promotes the clearance of parkin via the autophagy pathway, raising the intriguing possibility that increased turnover of parkin may contribute to the pathogenesis of MJD and help explain some of the Parkinsonian features in MJD. In addition to parkin, the *U*-box E3 ligase CHIP, a neuroprotective E3 implicated in protein quality control, was identified as a second E3 partner of ataxin-3, with ataxin-3 regulating the ability of CHIP to ubiquitinate itself. Indeed, ataxin-3 not only deubiquitinated CHIP, but also trimmed Ub conjugates on CHIP substrates, thereby regulating the length of Ub chains. Interestingly, when expanded ataxin-3 was present, CHIP levels were also reduced in the brains of MJD transgenic mice, raising the possibility that loss of one or both E3 partners may be a contributing factor in the pathogenesis of SCA3. In this review we discuss the implications from these studies and describe the importance of these findings in helping us understand the molecular processes involved in SCA3 and other neurodegenerative disorders.

## Ataxin-3: A Deubiquitinating Enzyme

Machado–Joseph disease (MJD) is one of nine polyQ disorders caused by a CAG expansion mutation within the *SCA3/MJD1* gene that encodes the *ataxin-3* protein (Kawaguchi et al., 1994). Expansion of its polyglutamine (polyQ) tract is believed to lead to a toxic gain of function, with calpain-dependent proteolysis of the mutant ataxin-3 generating expanded polyQ fragments and ultimately insoluble aggregates (Paulson et al., 1997; Fujigasaki et al., 2000; Chai et al., 2001; Koch et al., 2011). However, despite the shared mechanisms between these disorders, differences exist at both the clinical and neuropathological level, which cannot be accounted for by the polyQ expansion alone. Indeed, understanding the normal function of ataxin-3 can help explain why expansion of the polyQ tract in ataxin-3 is causing some of the specific features associated with MJD.

**Figure 1:**
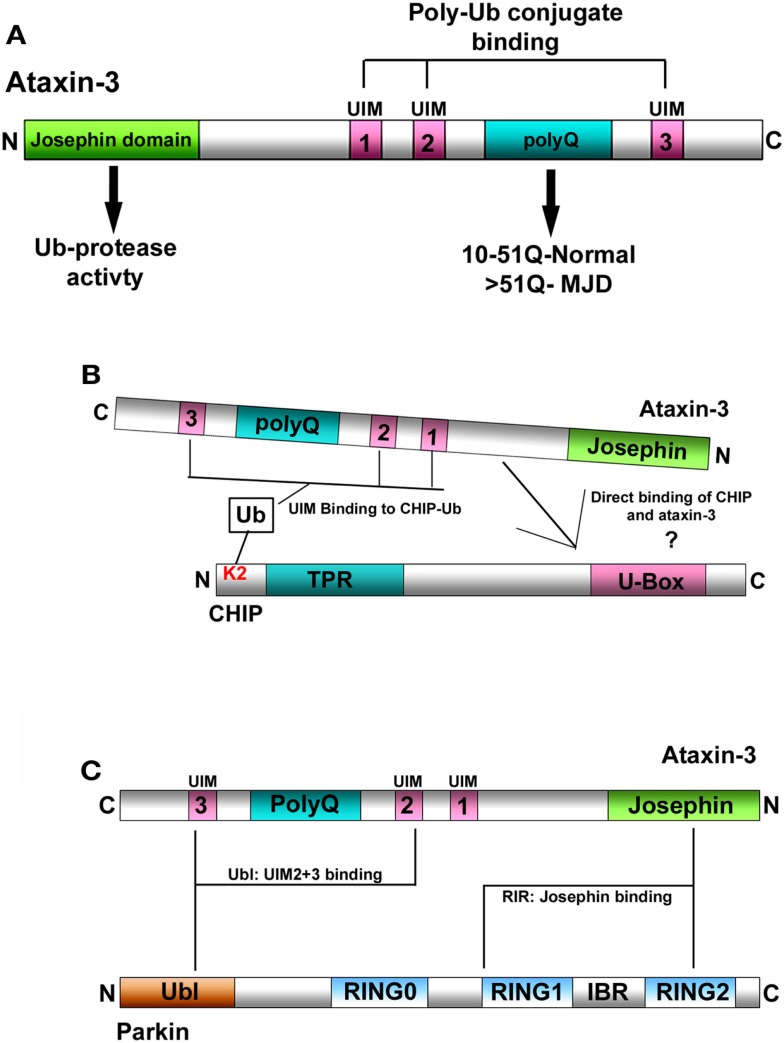
**(A)** Schematic representation of ataxin-3. Ataxin-3 contains an N-terminal Josephin domain, and three Ub-interacting motifs (UIMs) that flank a polyQ tract of variable length. In normal individuals, this tract can be anywhere from 10 to 51Q. In individuals with MJD, this tract has expanded to now contain >51Q. **(B)** Schematic representation of the interaction between ataxin-3 and CHIP. (1) CHIP contains an N-terminal tetratricopeptide repeat (TPR) domain and a C-terminal *U*-Box domain that confers CHIP with E3-Ub ligase activity. CHIP monoubiquitinates itself at lysine 2 (K2), which in turn facilitates the interaction between CHIP and ataxin-3. CHIP and ataxin-3 also interact directly although the nature of this interaction has yet to be characterized. **(C)** Schematic representation of the interaction between ataxin-3 and parkin. Parkin interacts directly with ataxin-3 via its N-terminal Ub-like domain (Ubl) that binds to the UIMs of ataxin-3, and through its inbetweenring-RING2 (IBR-RING2) domain binding to the Josephin domain of ataxin-3.

A well-characterized function of ataxin-3 is its role in the ubiquitin (Ub)-proteasome (UPS) system (Burnett et al., 2003; Doss-Pepe et al., 2003; Scheel et al., 2003) as a deubiquitinating enzyme (DUB). As a DUB, ataxin-3 possesses two distinct features for it to function in the UPS. The first is its N-terminal Josephin domain, that confers ataxin-3 with a cysteine protease activity for hydrolyzing Ub linkages (Scheel et al., 2003; Nicastro et al., 2005). The second critical feature are its three Ub-interacting motifs (UIMs), through which ataxin-3 binds Ub conjugates and ubiquitinated proteins (Burnett et al., 2003; Donaldson et al., 2003), bringing it into proximity to trim or edit specific linkages within these Ub conjugates (Burnett and Pittman, 2005; Winborn et al., 2008; Scaglione et al., 2011) (Figure 1A). Thus, ataxin-3 can bind to and deubiquitinate Ub conjugates, leading us to ask, what are the ubiquitinated substrates ataxin-3 acts upon. Moreover, what are the functional consequences of ataxin-3-mediated deubiquitination and does the expansion of the polyQ tract in MJD affect this normal function?

Ataxin-3 is one of 98 known DUBs, that function to deubiquitinate a wide range of substrates, that includes many E3-Ub ligases. E3s function within the UPS to mediate the covalent attachment of Ub onto lysine residues within target proteins (Shimura et al., 2000) thereby influencing many different cellular pathways. Typically, DUBs oppose this activity of the E3’s by mediating the removal of Ub from target proteins. However, many E3s regulate their own stability and ability by ubiquitinating themselves. Often, these Ub conjugates target the E3 to the proteasome for degradation (Kao et al., 2000), although destruction of the E3 can be delayed or prevented by one or more DUBs removing these conjugates (Nathan et al., 2008). Paradoxically, DUBs can sometimes promote the degradation of an E3 by removing non-canonical Ub conjugates, which are protecting the E3 from degradation, enabling another E3 to ubiquitinate the E3 in question, thereby promoting its degradation (de Bie et al., 2010). To complicate matters, certain DUBs have been demonstrated to regulate the activity of E3s via deubiquitination (Scaglione et al., 2011). Given that DUBs often deubiquitinate multiple E3s (Daviet and Colland, 2008; Nathan et al., 2008), and since the number of E3s far outnumber DUBs, it is likely that ataxin-3-mediated deubiquitination is regulating the function of multiple E3s.

## Ataxin-3 and CHIP

Early studies hinted that the *U*-box E3-Ub ligase CHIP might be one such E3 that interacts with ataxin-3. Consistent with this notion, CHIP was first observed to ubiquitinate the expanded form of ataxin-3, thereby directing it for degradation (Jana et al., 2005) In later studies, ataxin-3 was demonstrated to deubiquitinate CHIP, making CHIP the first E3 identified that could be deubiquitinated by ataxin-3 (Winborn et al., 2008). Conversely, CHIP ubiquitinates wild-type ataxin-3, which in turn enhances the overall deubiquitinating activity of ataxin-3 (Todi et al., 2010). Thus, CHIP and ataxin-3 interact and regulate the activity of each other. Of note, unlike other E3: DUB partners in which levels of the E3 are regulated by deubiquitination, changes in ataxin-3 levels do not have any effect on CHIP levels, making it unlikely that ataxin-3-mediated deubiquitination regulates degradation of CHIP (Scaglione et al., 2011). Rather, ataxin-3 appears to regulate the E3 ligase activity of CHIP in the protein quality control pathway.

Typically, E3-Ub ligases mediate the attachment of Ub moieties onto themselves, and CHIP is no exception. Through its interaction with the E2-conjugating enzyme Ube2W, CHIP robustly monoubiquitinates itself on lysine 2 (K2), which in turn enhances its overall E3 ligase activity (Scaglione et al., 2011). When CHIP was unable to self-ubiquitinate, its ability to ubiquitinate a variety of substrates was impaired. Intriguingly, monoubiquitination of CHIP appears to enhance the interaction between ataxin-3 and CHIP, and when bound, ataxin-3 can now deubiquitinate CHIP (Figure 1B). Evidence further supporting this notion that the ability of ataxin-3 to deubiquitinate CHIP is coupled to the ligase activity of CHIP, came from assays in which ataxin-3-mediated deubiquitination of CHIP did not occur until after poly-Ub conjugates on substrate proteins had attained a certain length. Once this occurred, ataxin-3 could now deubiquitinate both CHIP and its substrates, trimming the Ub conjugates at the distal ends. This prevented these conjugates from being extended further (Scaglione et al., 2011). Taken together, ataxin-3 opposes the activity of CHIP by deubiquitinating CHIP and by editing the Ub conjugates that it forms on different substrates.

As ataxin-3 tightly regulates the ability of CHIP to ubiquitinate itself and its substrates, why then might this ability of ataxin-3 to edit Ub conjugates be important? With CHIP, ataxin-3 ensures that the Ub conjugates are the appropriate length to efficiently target substrates for proteasomal degradation. Furthermore, by inactivating CHIP via deubiquitination, ataxin-3 tightly regulate the activity of CHIP within the protein quality control pathway, ensuring that it can only ubiquitinate misfolded proteins targeted for degradation. However, if CHIP is unable to remove these proteins, resulting in an accumulation of misfolded proteins, ataxin-3 again utilizes its editing activity to help sequester these proteins into structures termed aggresomes, thereby mitigating the effects of misfolded protein toxicity within the cell (Ouyang et al., 2012; Wang et al., 2012). In this pathway, ataxin-3 edits Ub conjugates on misfolded proteins to generate free Ub C termini that are recognized by HDAC6 and subsequently sequestrated to the aggresomes (Ouyang et al., 2012). Thus, as an editor of Ub conjugates, ataxin-3 not only tightly regulates CHIP in the protein quality control pathway, but acts independently of CHIP to sequester misfolded proteins into aggresomes, providing the cell with multiple layers of protection from misfolded protein toxicity.

## Ataxin-3-Mediated Regulation of Parkin Activity

Individual DUBs often deubiquitinate and regulate the activity of multiple E3s (Daviet and Colland, 2008; Nathan et al., 2008), making it likely that ataxin-3 can deubiquitinate other E3s. Interestingly, MJD can present with clinical and neuropathological symptoms of Parkinson disease (PD), and this raises the possibility that an interaction between ataxin-3 and a PD-associated protein could be involved in MJD (Gwinn-Hardy et al., 2001; Bettencourt et al., 2011). Similar to CHIP, the PD-associated E3 parkin was demonstrated to ubiquitinate and facilitate the clearance of an expanded ataxin-3 fragment (Tsai et al., 2003). Parkin interacts directly with distinct UIMs in ataxin-3 via its N-terminal Ub-like domain (Ubl^parkin^) (Figure 1C) (Durcan et al., 2011). Through its interaction with parkin, ataxin-3 regulates the ability of parkin to ubiquitinate itself, with ataxin-3 reducing parkin self-ubiquitination both in cells and *in vitro*. Interestingly, although ataxin-3 deubiquitinates parkin, parkin is unable to ubiquitinate ataxin-3 (Durcan et al., 2011, 2012). Finally, ataxin-3-mediated deubiquitination appears to only regulate parkin activity, as the presence or absence of wild-type ataxin-3 had no effect on overall parkin levels (Durcan et al., 2011).

With CHIP, ataxin-3 could trim/edit Ub chains after they had formed on substrates and was able to catalyze the removal of mono-Ub off CHIP (Scaglione et al., 2011). With parkin, ataxin-3 was unable to remove individual Ub moieties or preformed Ub chains after they had formed, suggesting that ataxin-3 was deubiquitinating parkin through a more unconventional mechanism (Durcan et al., 2012). One clue came from *in vitro* assays in which ataxin-3 regulates the formation of Ub conjugates on parkin, only when parkin was actively ubiquitinating. These findings suggest that ataxin-3 actively opposes the ligation of Ub on parkin, thereby impeding the ability of parkin to self-ubiquitinate.

**Figure 2:**
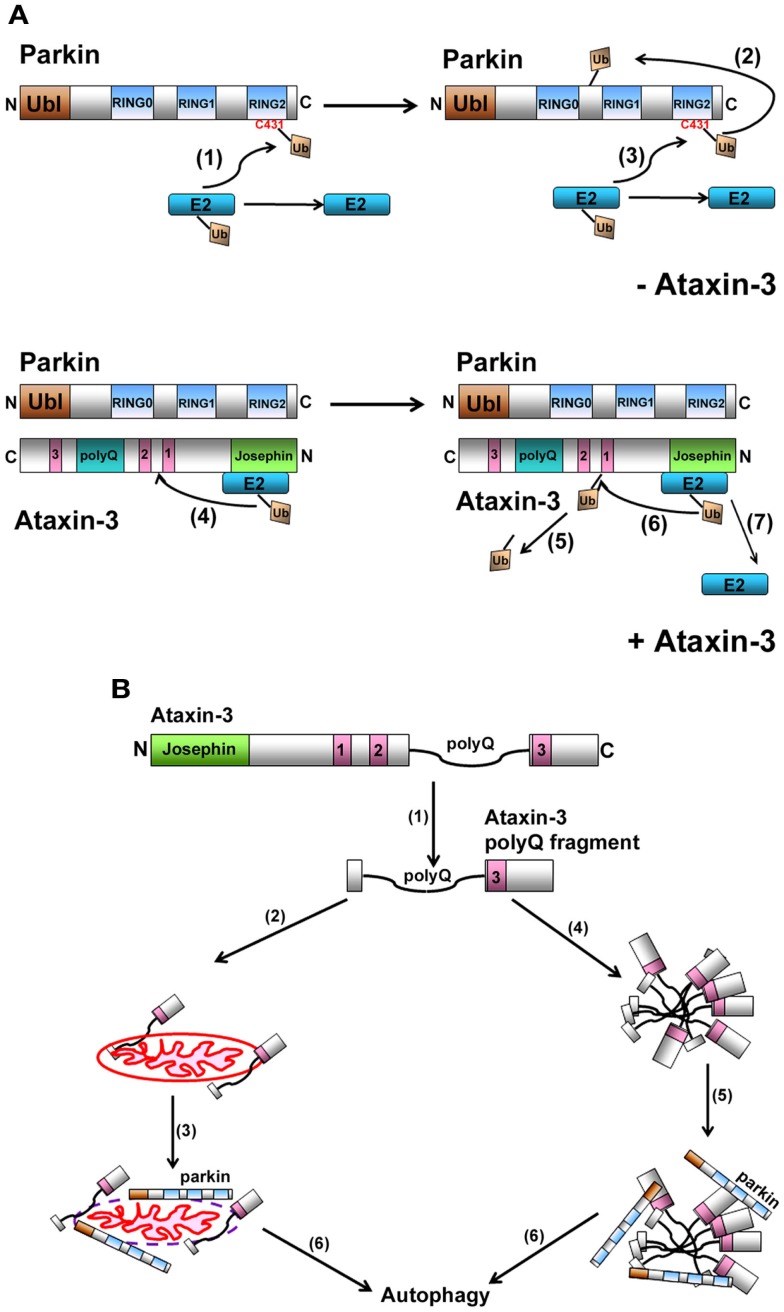
**(A)** Model representation of how ataxin-3-mediated deubiquitination regulates parkin self-ubiquitination. When parkin ubiquitinates itself, the charged E2-Ub thioester transfers the Ub onto C431 in parkin to form a transient parkin-Ub thioester complex (1). Parkin now transfers this Ub onto one of its own lysines generating an isopeptide linkage (2). Following the transfer of Ub onto itself, parkin is now free to receive a new Ub and this continues the cycle of parkin self-ubiquitination (3). When ataxin-3 is present, we propose that ataxin-3, through its interaction with the IBR-RING2 domain of parkin, blocks the C431 residue, impeding parkin from forming a parkin-Ub thioester. Instead, ataxin-3 now interacts with the E2, directing the transfer of Ub onto a lysine within ataxin-3 (4). This isopeptide linkage is transient as ataxin-3 can catalyze its removal (5) (unpublished data), allowing ataxin-3 to repeat this cycle of self-ubiquitination/deubiquitination, thereby reducing the ability of parkin to ubiquitinate itself (6 and 7). **(B)** Proposed model for how the expanded ataxin-3 can promote the autophagic clearance of parkin. In the presence of calcium, calpains cleave full-length ataxin-3, generating fragments containing the expanded polyQ tract (1). These fragments can associate with mitochondria (2), causing mitochondrial damage, parkin recruitment (3), and ultimately parkin-mediated clearance of itself and the mitochondria via the autophagy pathway (6). Alternatively, these expanded polyQ fragments also have a propensity to aggregate (4), recruiting parkin and CHIP (5), which now act to direct these aggregates and themselves for autophagic clearance (6).

Recently, parkin has been demonstrated to function as a RING/HECT hybrid, with the charged E2-Ub directly transferring the Ub onto cysteine 431 of parkin (Wenzel et al., 2011; Lazarou et al., 2013). This results in the formation of a parkin-Ub thioester intermediate complex, prior to parkin ligating the Ub onto itself (Figure 2A). Given that ataxin-3 and parkin interact directly, as demonstrated from *in vitro* binding data, how then is ataxin-3 interfering with the ability of parkin to ubiquitinate itself. Through its cysteine at residue 14, ataxin-3 alone can interact directly with the E2s used by parkin to self-ubiquitinate (Durcan et al., 2012). Remarkably, when the cysteine was mutated to a serine, this interaction was now abolished. When parkin was present, ataxin-3 could not only interact with both parkin and the E2, but it could also promote the transfer of the Ub away from parkin and onto itself. Such E3-like activity is not unprecedented for a DUB, with both UCH-L1 and A20 possessing E3 and DUB activities (Liu et al., 2002; Wertz et al., 2004), although further work is required to ascertain how ataxin-3 might act as an E3-like DUB. From these findings, we propose a model whereby ataxin-3 binds directly to parkin, blocking the E2 from transferring the Ub onto C431 in parkin. As a result, the E2 now transfers the Ub onto ataxin-3 and away from parkin, thereby impeding the activity of parkin to ubiquitinate itself.

## MJD, Mutant Ataxin-3, and Its E3 Partners

From these studies, it is clear that ataxin-3 is involved in tightly regulating the activity of two E3s that function to maintain normal cellular homeostasis. Strikingly, in mouse models of MJD, levels of both CHIP and parkin are significantly reduced when the polyQ tract becomes expanded over 51 glutamines, thereby disrupting the normal cellular homeostasis and promoting neuronal cell loss. Thus, when mutated, ataxin-3 now promotes the destruction of two quality control E3s. Yet, why does the presence of an expanded polyQ tract in ataxin-3 enhance the clearance of both E3s, is this effect dependent on the DUB ability of ataxin-3 or is it caused by the expanded polyQ tract. In the case of CHIP, expansion of the polyQ tract increases the binding between CHIP and ataxin-3, leading to a reduction in CHIP levels in the brains of MJD transgenic mice (Scaglione et al., 2011). One possibility is that the increased affinity of mutant ataxin-3 for CHIP somehow alters their functional relationship, inadvertently causing CHIP to be directed for degradation. Although it is unclear why CHIP is degraded, a reduction in CHIP levels will have an adverse effect on the protein quality control pathway. Consequently, an accumulation of misfolded neurotoxic proteins can be a key contributing factor in the progressive loss of neurons associated with MJD.

Intriguingly, the presence of the mutant ataxin-3 also causes a reduction in parkin levels in the brains of MJD transgenic mice. These findings were further confirmed and extended in cells, with the presence of the expanded form of ataxin-3 promoting clearance of parkin through the autophagy pathway (Durcan et al., 2011). However the mechanism remains unclear. Recent studies have highlighted the role of K27-, K29-, and K63-linked Ub in directing proteins for degradation via the lysosomal and autophagy pathways. Interestingly, parkin assembles Ub conjugates on itself preferentially via these linkages and when compared to wild-type ataxin-3, the polyQ expanded mutant ataxin-3 was more efficient at removing K27- and K29-linked Ub conjugates from parkin. One possibility is that these K27- and K29-linked Ub conjugates protect parkin from autophagic degradation, and their preferential removal enhances parkin turnover via autophagy (Durcan and Fon, 2011).

Alternatively, and a more likely scenario, is that the effect of expanded ataxin-3 on parkin occurs independently of its catalytic activity and is a direct effect of the expanded polyQ tract (Figure 2B). Autophagy has been implicated in clearing mutant polyQ protein aggregates in several models of polyQ expansion disorders, including MJD. In this scenario, fragments of the expanded polyQ tract generated from the mutant ataxin-3 cause aggregates to form. By interacting with these inclusions and directing them for destruction, CHIP and parkin may in a sense “just go along for the ride,” ultimately resulting in both their clearance along with the aggregates. Thus, over time, extensive clearance of these aggregates can cause levels of CHIP and parkin to diminish. We also cannot exclude a second possibility, that parkin and CHIP are actively involved in the autophagic process, as has been reported for the recently identified role for parkin in targeting damaged mitochondria for autophagic removal (mitophagy) (Narendra et al., 2008). Interestingly, mitochondrial abnormalities have been observed in models of polyQ disorders, including MJD (Ranganathan et al., 2009; Yu et al., 2009; Kazachkova et al., 2012; Laco et al., 2012; Reddy and Shirendeb, 2012). Moreover, polyQ fragments generated from mutant ataxin-3 can interact with and damage mitochondria (Pozzi et al., 2008; Sugiura et al., 2011; Kazachkova et al., 2012). This in turn can now potentially promote the mitochondria recruitment of parkin, triggering the autophagic clearance of parkin-bound mitochondria (Chai et al., 2001; Koch et al., 2011). Taken together, ataxin-3 forms partnerships with two E3s that are essential for maintaining normal cellular homeostasis. In MJD, these partnership are not only disrupted, but the presence of the expanded ataxin-3 now promotes clearance of both parkin and CHIP, which over time can have deleterious consequences on neurons in MJD and PD.

## Conflict of Interest Statement

The authors declare that the research was conducted in the absence of any commercial or financial relationships that could be construed as a potential conflict of interest.
